# Effective treatment of severe COVID-19 patients with tocilizumab

**DOI:** 10.1073/pnas.2005615117

**Published:** 2020-04-29

**Authors:** Xiaoling Xu, Mingfeng Han, Tiantian Li, Wei Sun, Dongsheng Wang, Binqing Fu, Yonggang Zhou, Xiaohu Zheng, Yun Yang, Xiuyong Li, Xiaohua Zhang, Aijun Pan, Haiming Wei

**Affiliations:** ^a^Respiratory and Critical Care Medicine, The First Affiliated Hospital of University of Science and Technology of China (Anhui Provincial Hospital), Hefei, Anhui 230000, People’s Republic of China;; ^b^Respiratory and Critical Care Medicine, Anhui Fuyang Second People’s Hospital, Fuyang, Anhui 230000, People’s Republic of China;; ^c^Institute of Immunology and the Chinese Academy of Sciences (CAS) Key Laboratory of Innate Immunity and Chronic Disease, School of Life Science and Medical Center, University of Science and Technology of China, Hefei, Anhui 230000, People’s Republic of China;; ^d^Hefei National Laboratory for Physical Sciences at Microscale, University of Science and Technology of China, Hefei, Anhui 230000, People’s Republic of China;; ^e^Intensive Care Unit, The First Affiliated Hospital of University of Science and Technology of China (Anhui Provincial Hospital), Hefei, Anhui 230000, People’s Republic of China;; ^f^Hemodialysis Center, Anhui Fuyang Second People’s Hospital, Fuyang, Anhui 236000, People’s Republic of China

**Keywords:** tocilizumab, interleukin-6, COVID-19, SARS-CoV-2, cytokine storm

## Abstract

After analyzing the immune characteristics of patients with severe coronavirus disease 2019 (COVID-19), we have identified that pathogenic T cells and inflammatory monocytes with large amount of interleukin 6 secreting may incite the inflammatory storm, which may potentially be curbed through monoclonal antibody that targets the IL-6 pathways. Here, we aimed to assess the efficacy of tocilizumab in severe patients with COVID-19 and seek a therapeutic strategy. The patients diagnosed as severe or critical COVID-19 in The First Affiliated Hospital of University of Science and Technology of China (Anhui Provincial Hospital) and Anhui Fuyang Second People’s Hospital were given tocilizumab in addition to routine therapy between 5 and 14 February 2020. The changes of clinical manifestations, computerized tomography (CT) scan image, and laboratory examinations were retrospectively analyzed. Fever returned to normal on the first day, and other symptoms improved remarkably within a few days. Within 5 d after tocilizumab, 15 of the 20 patients (75.0%) had lowered their oxygen intake, and 1 patient needed no oxygen therapy. CT scans manifested that the lung lesion opacity absorbed in 19 patients (90.5%). The percentage of lymphocytes in peripheral blood, which decreased in 85.0% of patients (17/20) before treatment (mean, 15.52 ± 8.89%), returned to normal in 52.6% of patients (10/19) on the fifth day after treatment. Abnormally elevated C-reactive protein decreased significantly in 84.2% of patients (16/19). No obvious adverse reactions were observed. All patients have been discharged on average 15.1 d after giving tocilizumab. Preliminary data show that tocilizumab, which improved the clinical outcome immediately in severe and critical COVID-19 patients, is an effective treatment to reduce mortality.

## Significance

In patients with coronavirus disease 2019, a large number of T lymphocytes and mononuclear macrophages are activated, producing cytokines such as interleukin-6 (IL-6), which bind to the IL-6 receptor on the target cells, causing the cytokine storm and severe inflammatory responses in lungs and other tissues and organs. Tocilizumab, as a recombinant humanized anti-human IL-6 receptor monoclonal antibody, can bind to the IL-6 receptor with high affinity, thus preventing IL-6 itself from binding to its receptor, rendering it incapable of immune damage to target cells, and alleviating the inflammatory responses.

In the past decades, two known zoonotic coronaviruses, severe acute respiratory syndromes coronavirus (SARS-CoV) and Middle East respiratory syndrome coronavirus (MERS-CoV), have been reported to damage the respiratory tract and cause severe outbreaks (1–3). Severe acute respiratory syndrome coronavirus 2 (4) is a newly discovered coronavirus, which was first discovered in Wuhan, China in December 2019. The disease was officially named coronavirus disease 2019 (COVID-19) on 11 February 2020 (5). Epidemiological data have basically determined the route of person-to-person transmission in COVID-19 (6, 7), which spread rapidly and has become a worldwide public health challenge. Most of the patients developed pneumonia, which can worsen rapidly into respiratory failure (8). The elderly and patients with low immune function have a higher susceptibility and mortality (1). One study reported that patients usually have pneumonia with abnormal findings on chest computerized tomography (CT) scan (9). Common symptoms at onset include fever, cough, and myalgia or fatigue. A large number of patients experienced severe complications including acute respiratory distress syndrome (ARDS; 29%); 32% of patients needed an intensive care unit (ICU) admission, and six (15%) died (9). In another report of 99 cases, 17 (17%) patients developed ARDS, of which 11 (11%) worsened within a few days and died (7). According to a new report, the mortality for critical cases reached 60.5% (10). Unfortunately, the pathogenesis of COVID-19 still remains unclear, and there are no efficient therapeutics.

Study demonstrated that in the pathogenesis of severe acute respiratory syndrome (SARS), a cytokine storm occurred, involving a considerable release of proinflammatory cytokine including interleukin-6 (IL-6), tumor necrosis factor-α (TNF-α), and IL-12 (11). In the research of Middle East respiratory syndrome (MERS), caused by another coronavirus (MERS-CoV), cytokine genes of IL-6, IL-1β, and IL-8 can be markedly high. A delayed proinflammatory cytokine induction by MERS-CoV was also confirmed (12). Similar to the changes in SARS and MERS, in COVID-19, higher plasma levels of cytokines including IL-6, IL-2, IL-7, IL-10, granulocyte-colony stimulating factor, interferon-γ (IFN-γ)–inducible protein, monocyte chemoattractant protein, macrophage inflammatory protein 1α, and TNF-α were found in ICU patients, which implied that a cytokine storm occurred (7, 9) and related to the severity and prognosis of the disease. In the biopsy samples at autopsy from a patient who died from the severe infection with COVID-19, histological examination showed bilateral diffuse alveolar damage with cellular fibromyxoid exudates. Mononuclear inflammatory lymphocytes were seen in both lungs (13). These studies suggested that an inflammatory factor or a cytokine storm had occurred. In our previous research, after analyzing the immune characteristics of patients with COVID-19, we have found that aberrant pathogenic T cells and inflammatory monocytes with a large number of cytokines secreting may incite an inflammatory storm. This research has found flow cytometric profiles of increased IL-6, granulocyte-macrophage colony stimulating factor (GM-CSF), and IFN-γ in these severe patients, whereas TNF-α was not significantly up-regulated in CD4^+^ T cells from COVID-19 patients (14). So, we believe that IL-6 and GM-CSF are the key cytokines leading to inflammatory storm, which may result in increased alveolar–capillary blood–gas exchange dysfunction, especially impaired oxygen diffusion, and eventually lead to pulmonary fibrosis and organ failure. Therefore, we suggested that IL-6 might play a key role in the cytokine storm, and interfering of IL-6 might be a potentially therapeutic for severe and critical COVID-19.

IL-6 receptor (IL-6R) has two forms: membrane-bound interleukin-6 receptor (mIL-6R) and soluble interleukin-6 receptor (sIL-6R). IL-6 binds to sIL-6R to form a complex, which then binds to gp130 on the cell membrane to complete transsignal transduction and play a proinflammatory role (15–18). As a recombinant humanized anti-human IL-6 receptor monoclonal antibody, tocilizumab can specifically bind sIL-6R and mIL-6R and inhibit signal transduction. It is currently used mainly for rheumatoid arthritis (18). The results of long-term toxicity tests on animals showed that tocilizumab was well tolerated, and no significant abnormalities were observed in other clinicopathological studies or histopathological evaluations (18–20). In this study, we prospectively observed tocilizumab in treating severe or critical COVID-19 patients using historical data to see if IL-6 plays a pivot role in the pathogenesis and the efficacy of the tocilizumab interference of IL-6 in order to provide a therapeutic strategy for this fatal disease.

## Results

### Demographic Characteristics.

The average age of the subjects was 56.8 ± 16.5 y and ranged from 25 to 88 y (Table 1). Of the 21 patients, 18 were males (85.7%), and 3 were females (14.3%); 17 patients (81.0%) were assessed as severe, and 4 (19.0%) were critical. Among them, five patients (23.8%) had a history of exposure to Wuhan, and six (28.6%) had exposure to patients who had confirmed COVID-19. Others had no epidemiological history when questioned. Eighteen patients (85.7%) received tocilizumab once, and 3 patients (14.3%) had another one at the same dose due to fever within 12 h.

**Table 1.** Demographic characteristics of the patients on presentation

Characteristic	Patients (*n* = 21)
Age (range), y	56.8 ± 16.5 (25–88)*
Gender	
Male	18/21 (85.7%)
Female	3/21 (14.3%)
Chronic medical illness	
Hypertension	9/21 (42.9%)
Diabetes	5/21 (23.8%)
CHD	2/21 (9.5%)
COPD	1/21 (4.8%)
Brain infarction	1/21 (4.8%)
Bronchiectasis	1/21 (4.8%)
Auricular fibrillation	1/21 (4.8%)
CKD	1/21 (4.8%)
Exposure	
Exposure to Wuhan	5/21 (23.8%)
Exposure to patients^†^	6/21 (28.6%)
Symptoms	
Fever	21/21 (100%)
Cough	14/21 (66.7%)
Phlegm	9/21 (42.9%)
Fatigue	6/21 (28.6%)
Chest tightness	6/21 (28.6%)
Nausea	4/21(19.0%)
Rhinorrhea	1/21 (4.8%)
Chest pain	1/21 (4.8%)
Body temperature, °C	38.8 ± 0.6 (37.6–40.6)
37.6–38.6	11/21 (52.4%)
38.6–39.6	8/21 (38.1%)
39.6–40.6	2/21 (9.5%)
Time to progression, d	5.6 ± 2.8 (2–14)*
Laboratory tests before tocilizumab^‡^	
Neutrophils percentage (%, 40–75)	82.28 ± 9.14 (16/20, 80.0%)*
Platelet count (×10^9^/L, 125–350)	170.35 ± 58.26 (4/20, 20.0%)*
Erythrocyte sedimentation rate (mm/h, 0–15)	42.42 ± 27.82 (10/12, 83.3%)*
d-dimer (μg/mL, 0–1.10)	0.80 ± 0.92 (2/19, 10.5%)*
Prothrombin time (s, 10.5–14.5)	12.39 ± 2.12 (4/19, 21.1%)*
Alanine aminotransferase (IU/L, 0–50)	29.55 ± 14.44 (1/20, 5.0%)*
Aspartate aminotransferase (IU/L, 15–40)	31.15 ± 9.25 (1/20, 5.0%)*
Creatine kinase (IU/L, 50–310)	162.41 ± 74.77 (1/19, 5.3%)*
Lactate dehydrogenase (U/L, 120–250)	370.70 ± 140.21 (17/20, 85.0%)*
Creatinine (μmol/L, 35–115)	78.20 ± 26.46 (2/20, 10.0%)*
Blood urea nitrogen (mmol/L, 3.1–8.0)	5.64 ± 2.75 (2/20, 10.0%)*
State of illness	
Severe	17/21 (81.0%)
Critical	4/21 (19.0%)
Oxygen therapy	
High-flow oxygen	9/20 (45.0%)
Nasal cannula	7/20 (35.0%)
Invasive ventilation	2/20 (10.0%)
Noninvasive ventilation	1/20 (5.0%)
Mask oxygen	1/20 (5.0%)
Tocilizumab adverse events	0
Clinical outcome	
Discharge from hospital	21/21 (100%)
Hospitalization days (range), d^§^	15.1 ± 5.8 (10–31)*
≤14	13/21 (61.9%)
14–21	6/21 (28.6%)
≥21	2/21 (9.5%)

Data are *n*/*N* (%) unless specified otherwise. CHD, coronary heart disease; CKD, chronic kidney disease; COPD, chronic obstructive pulmonary disease.

* Plus–minus values are means ± SD.

^†^ Patients who have confirmed COVID-19.

^‡^ Not all patients received all relevant laboratory tests.

^§^ Hospitalization days after the treatment with tocilizumab.

### Clinical Presentations.

All patients presented with fever as the first symptom followed by dry cough (14/21, 66.7%), a small amount of white phlegm (9/21, 42.9%), fatigue (6/21, 28.6%), and chest tightness (6/21, 28.6%). Four (19.0%) patients had nausea. Other symptoms, including rhinorrhea and chest pain, were rare (4.8%). Among all patients, there was a mean time of 5.6 ± 2.8 d (range, 2 to 14) from the onset of fever to the occurrence of dyspnea. Twenty patients were treated with an oxygen therapy, including high-flow oxygen therapy in 9 patients (45.0%), nasal cannula in 7 patients (35.0%), mask oxygen in 1 patient (5.0%), noninvasive ventilation in 1 patient (5.0%), and invasive ventilation in 2 patients (10.0%). Also, one patient refused oxygen therapy. (Table 1)

### Laboratory Examinations.

Tables 1 and 2 show the results of baseline laboratory tests before tocilizumab. White blood cell count showed that four patients (4/20, 20.0%) have an abnormal value (mean, 6.30 ± 2.77 × 10^9^/L) in peripheral blood. The percentage of lymphocytes was decreased in 85.0% of patients (17/20; mean, 15.52 ± 8.89%); 80.0% of patients (16/20) have elevated percentage of neutrophils (mean, 82.28 ± 9.14%). Ten of 12 patients (83.3%) had elevated erythrocyte sedimentation rate (mean, 42.42 ± 27.82 mm/h). The mean of d-dimer was 0.80 ± 0.92 μg/mL, and prothrombin time was 12.39 ± 2.12 s. C-reactive protein (CRP) levels increased in all 20 patients (mean, 75.06 ± 66.80 mg/L). The mean of procalcitonin (PCT) value was 0.33 ± 0.78 ng/mL, and only 2 of 20 patients (10.0%) presented an abnormal value. One patient (1/19, 5.3%) had an elevated creatine kinase with a mean of 162.41 ± 74.77 IU/L. Lactate dehydrogenase levels increased in 85.0% of patients (17/20; mean, 370.70 ± 140.21 U/L). Only 1 of 20 patients (5.0%) had slightly elevated alanine aminotransferase and aspartate aminotransferase (means, 29.55 ± 14.44 and 31.15 ± 9.25 IU/L, respectively). Creatinine and blood urea nitrogen increased slightly in two patients (2/20, 10%), with mean values of 78.20 ± 26.46 μmol/L and 5.64 ± 2.75 mmol/L, respectively.

**Table 2.** Laboratory tests before and after tocilizumab

	Normal range	Before tocilizumab	After tocilizumab		
			D1	D3	D5
White cell count, ×10^9^/L	3.5–9.5	6.30 ± 2.77 (4/20, 20.0%)	8.05 ± 4.39 (8/18, 44.4%)	6.02 ± 3.05 (9/21, 42.9%)	5.25 ± 2.11 (2/19, 10.5%)
Lymphocyte percentage, %	20–50	15.52 ± 8.89 (17/20, 85.0%)	11.78 ± 11.36 (16/18, 88.9%)	16.93 ± 13.59 (14/21, 66.7%)	22.62 ± 13.48 (9/19, 47.4%)
CRP, mg/L	0–5	75.06 ± 66.80 (20/20, 100%)	38.13 ± 54.21 (17/18, 94.4%)	10.61 ± 13.79 (10/20, 50.0%)	2.72 ± 3.60 (3/19, 15.8%)
Procalcitonin, ng/mL	0–0.5	0.33 ± 0.78 (2/20, 10.0%)	0.21 ± 0.35 (2/16, 12.5%)	0.09 ± 0.13 (1/19, 5.3%)	0.12 ± 0.15 (1/18,5.6%)
IL-6, pg/mL	0–7	153.44 ± 296.63 (18/18, 100%)	129.18 ± 131.79 (13/13, 100%)	300.98 ± 341.90 (17/17, 100%)	274.90 ± 414.08 (12/12, 100%)

Data are means ± SD (abnormal no./total no., %). D, day after tocilizumab.

Additionally, all patients had been analyzed for IL-6 expression levels before tocilizumab. The mean of 18 patients tested by electrochemical luminescence method was 153.44 ± 296.63 pg/mL. The remaining three patients from Anhui Provincial Hospital have analyzed their IL-6 expression in peripheral blood lymphocyte with 6.95, 7.40, and 7.72% IL-6^+^ cells of CD4^+^ T cells. Both methods have shown IL-6 up-regulation in these severe and critical COVID-19 patients.

### Imaging Features.

**Fig. 1.**
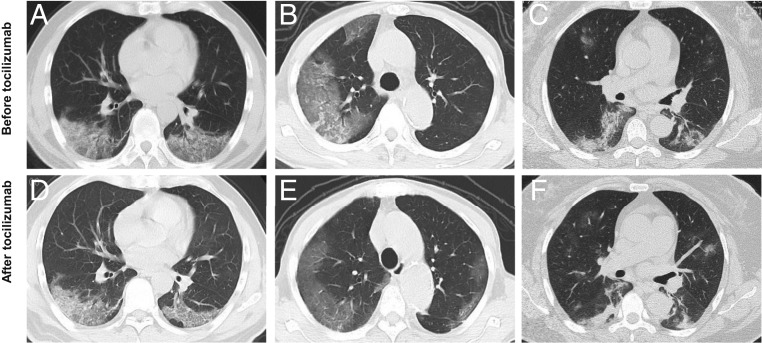
Chest CT scans showed significant remission in both lungs in patients after the treatment with tocilizumab. (*A*–*C*) Computerized tomography (CT) showed plaque-like and ground glass opacities before the treatment with tocilizumab. (*D*–*F*) Chest CT showed diffuse infiltration in both lungs, but the lesions were clearly absorbed after the treatment with tocilizumab.

All patients had abnormal chest CT on presentation. The primary abnormality on the initial chest CT was plaque-like, ground glass opacities and focal consolidation, mainly distributed in the peripheral, especially the subpleural region (Fig. 1 *A*–*C*). The ground glass opacities increased in size, extent, and severity in 21 patients within the first 7 d after admission before receiving tocilizumab. No pleural effusions, mediastinal nodes, or central pulmonary emboli were found.

### Treatment Outcomes.

**Fig. 2.**
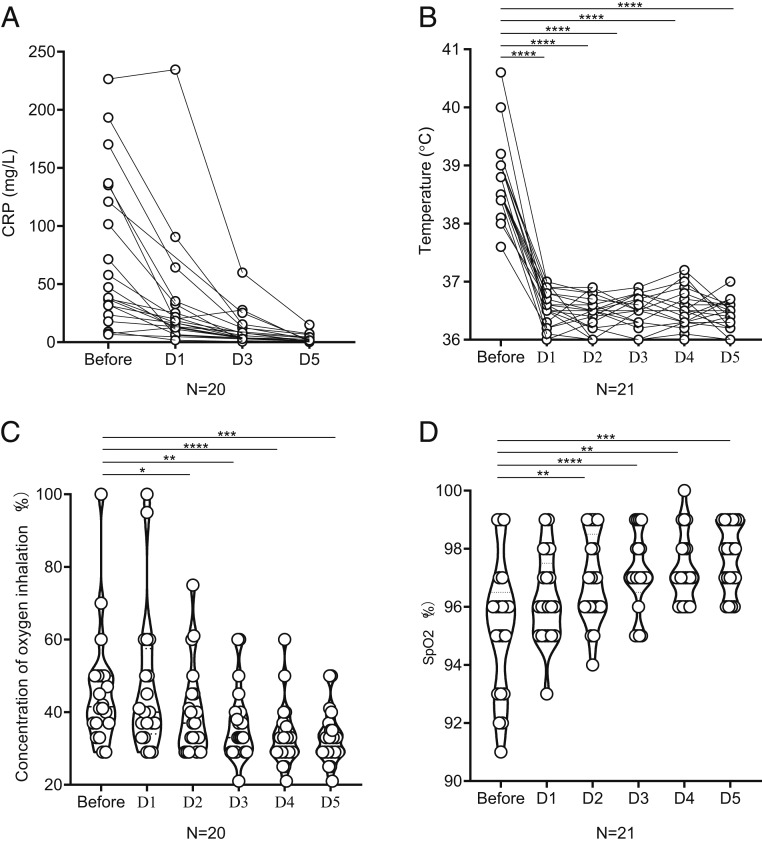
The values of CRP, body temperature, concentration of oxygen inhalation, and oxygen saturation before and after the treatment with tocilizumab for the 21 patients with COVID-19. (*A*) CRP decreased significantly after the treatment with tocilizumab and returned to normal in the majority of the patients. (*B*) The fever returned to normal in all 21 patients after tocilizumab. (*C* and *D*) Before the treatment, 20 patients needed oxygen therapy except 1 who refused. After tocilizumab, 15 patients had lowered their oxygen intake, and the oxygen saturation remained stable. Among them, one patient did not need further oxygen therapy on the third day. Therefore, the concentration of his oxygen inhalation during D3-D5 has marked as 21%, similar as oxygen content in normal air. (*B*–*D*) *P < 0.05, **P < 0.01, ***P < 0.005, ****P < 0.0001. D, day after tocilizumab; SpO2, percutaneous blood oxygen saturation.

The body temperature of all patients dramatically returned to normal on the first day after receiving tocilizumab and remained stable thereafter (Fig. 2*B*). Clinical symptoms were significantly relieved synchronously in the following days. The peripheral oxygen saturation improved remarkably (Fig. 2 *C* and *D*). Fifteen patients (15/20, 75.0%) had lowered their oxygen intake within 5 d after the treatment with tocilizumab. Among these patients, one patient did not need further oxygen therapy. Among the three patients who used ventilator, one patient was taken off the noninvasive ventilator on the first day after tocilizumab, one patient had tracheal extubated and regained consciousness on the fifth day and another one on the eleventh day.

A significant change of the percentage of lymphocytes and CRP levels was observed after tocilizumab treatment, as shown in Fig. 2*A* and Table 2. On the fifth day after treatment, only two patients (2/19, 10.5%) had an abnormal value in white blood cell count with a mean of 5.25 ± 2.11 × 10^9^/L. The percentage of lymphocytes in 10 patients (10/19, 52.6%) returned to normal (mean, 22.62 ± 13.48%). CRP decreased significantly and returned to normal in 84.2% of patients (16/19; mean, 2.72 ± 3.60 mg/L) after treatment on the fifth day. The value of IL-6 did not decrease significantly in the short term after treatment with tocilizumab (Table 2). After treatment, CT scans showed that the lesions were absorbed in 19 (90.5%) patients and a little improvement in the others (Fig. 1 *D*–*F*). All patients have been discharged including critical ones. Two consecutive negative viral qualitative tests with a time interval of 24 h have indicated that the viral load was completely cleared. The mean hospitalization time was 15.1 ± 5.8 d after the treatment with tocilizumab. Among them, 13 (61.9%) patients were discharged within 2 wk after tocilizumab, and 6 were discharged within 3 wk (Table 1).

### Safety.

All reported adverse events of patients were recorded. In our study, there were no serious events caused by tocilizumab. Adverse drug reactions, including elevated transaminase, neutropenia, infection, etc., were not reported. No emerging bacterial, fungal, or viral infections were observed during the treatment.

## Discussion

In this study, we retrospectively observed tocilizumab, an IL6R inhibitor, in treatment of 21 patients with severe and critical COVID-19. Clinical data showed that the symptoms, hypoxygenmia, and CT opacity changes were improved immediately after the treatment with tocilizumab in most of the patients, suggesting that tocilizumab could be an efficient therapeutic for the treatment of COVID-19.

COVID-19 is a newly occurred infectious respiratory disease with mild symptoms in the early stages of infection. However, in a considerable number of patients, the symptoms deteriorate rapidly and are manifested as chest tightness, shortness of breath, and even respiratory failure. CT scans often show rapid enlarged opacities. These patients usually need oxygen therapy and even assistant ventilation in the ICU. Unfortunately, 4.3 to 11% of them died even after recommended standard treatment (7, 8), as no effective therapeutics could be achieved. Additionally, the mortality for critical cases reached as high as 60.5% (10). In this research, all 21 patients had a history of routine treatment for a week before tocilizumab but deteriorated with sustained fever, hypoxygenmia, and CT image worsening. After the treatment with tocilizumab, in addition to the improvement of body temperature, the respiratory function was improved to some degree in most of the patients. Chest tightness was relieved. Most patients lowered their oxygen intake flow, and the oxygen saturation remained stable. Two patients were taken off the ventilator within 5 d. Therefore, it is necessary to start tocilizumab treatment as soon as possible when the following conditions occurred, including persistent fever, the condition changes from mild to severe (including high-risk factor for severe cases), diffuse lung opacities on CT scans, and elevated level of IL-6. Early treatment can effectively control the deterioration of symptoms.

The decrease of the percentage of lymphocytes has been considered an important indicator for diagnosis and severity judgment in COVID-19 patients (8). In our study, a lower percentage of lymphocytes was found in 85.0% of patients (17/20) and returned to normal in 52.6% of patients (10/19) within 5 d when tocilizumab was given. At the same time, elevated CRP also returned to normal. The level of IL-6 increased in all patients before treatment. After the treatment, the IL-6 levels will be temporarily increased in serum in the next few days, for its receptors have been blocked by tocilizumab. Similar conditions can be observed in chimeric antigen receptor T cell treatment (21). The lung opacities on CT scans were absorbed in 19 (90.5%) patients. Considering that the lung tissue damage needs sufficient time for repairing, remission delay in CT scan can be anticipated. During the treatment, no adverse drug reactions and subsequent pulmonary infections were reported. Clinical symptoms of all patients improved remarkably with good prognosis after the treatment. All patients have been discharged, and most of them were within 2 wk after tocilizumab. Therefore, tocilizumab can effectively treat severe patients of COVID-19, which might be explained by the blocking of IL-6–associated febrile and inflammatory storm response.

Nevertheless, there are several shortcomings in this study. The number of patients was rather limited. It was a single observation study, and a significant bias could have possibly existed. Apparently, the evidence strength needs to be enhanced. To get more evidence, a randomized, controlled trial and a study on the mechanism of IL-6 in COVID-19 are being under performing.

## Conclusion

In summary, tocilizumab effectively improve clinical symptoms and represses the deterioration of severe COVID-19 patients. Therefore, tocilizumab is an effective treatment in severe patients of COVID-19, which provided a therapeutic strategy for this fatal infectious disease.

## Methods

### Patients.

A total of 21 patients met the study conditions and were treated with tocilizumab between 5 and 14 February 2020. Seven of the patients were treated in The First Affiliated Hospital of University of Science and Technology of China (Anhui Provincial Hospital), and 14 patients were treated in Anhui Fuyang Second People’s Hospital. All patients enrolled met the severe or critical criteria defined by the “Diagnosis and Treatment Protocol for Novel Coronavirus Pneumonia (7th Interim Edition)” sponsored by the National Health Commission of the People’s Republic of China (22). For diagnosis, specimens were obtained by throat swabs under aseptic operation and tested with real-time RT-PCR assay that was developed from the publicly released virus sequence. The diagnosis of severity was defined if any of the following conditions were met: 1) respiratory rate ≥30 breaths per 1 min, 2) SpO2 ≤ 93% while breathing room air, 3) PaO_2_/FiO_2_ ≤ 300 mm Hg. A critical case was diagnosed if any of the following conditions were met: 1) respiratory failure, which requiring mechanical ventilation; 2) shock; 3) combined with other organ failure, need to be admitted to the ICU. Also, patients with active pulmonary tuberculosis combined with clear bacterial infection and fungal infection were excluded.

The Medical Research Ethics Committee of the Anhui Provincial Hospital approved the study. All patients signed informed consent before using tocilizumab and agreed to publish this case series. We are committed to protecting patient privacy and complying with the Helsinki Declaration [approval no. 2020-XG(H)-015].

### IL-6 Test.

The value of IL-6 was measured by electrochemical luminescence method (Roche Diagnostics GmbH) in 18 patients or fluorescence-activated cell sorting analysis in 3 patients. Intracellular staining of IL-6 was performed without adding any restimulation. The cells were then collected, washed, and blocked according to the instructions of eBioscience. The normal range of IL-6 in peripheral blood is less than 7 pg/mL.

### Treatment and Observation.

All patients received standard care as follows according to the “Diagnosis and Treatment Protocol for Novel Coronavirus Pneumonia (7th Interim Edition)” (22): 1) antiviral therapy of lopinavir/ritonavir (200/50 mg per tablet for adults twice a day, two tablets each time, and the course of treatment does not exceed 10 d), IFN-α (5 million U each time for adults or equivalent dissolved in 2 mL of sterilized water and aerosol inhalation twice a day), and ribavirin (recommended for use with IFN or lopinavir/ritonavir, 500 mg per dose for adults, intravenous [i.v.] drip two to three times a day, and the course of treatment does not exceed 10 d); 2) glucocorticoid (use for a short period of time, range 3 to 5 d, as appropriate, at a dose not exceeding the equivalent of 1 to 2 mg/kg per day methylprednisolone for patients with rapid progress in respiratory function and imaging and excessive activation of the inflammatory response); 3) other symptom relievers and oxygen therapy.

Also, they were treated with tocilizumab (Roche Pharma [Schweiz] Ltd; B2084B21).The first dose was 4–8 mg/kg body weight, and the recommended dose was 400 mg through an i.v. drip up to a maximum of 800 mg. Dilution was to 100 mL with 0.9% normal saline, and the infusion time was more than 1 h. In case of fever within 12 h, an additional dose was given (same as before), and the cumulative dose could not be more than two times.

Clinical features including body temperature, concentration of oxygen inhalation, and oxygen saturations were recorded daily before and after treatment. A whole-blood white cell count was performed repeatedly. All patients had been spiral CT scanned on admission and a week later after the beginning of tocilizumab treatment using a 64-row spiral Optima CT680 scanner (GE Healthcare) in a whole-lung, low-dosage exposure, scanning with 5-mm slices.

### Data Collection.

Clinical data were retrospectively analyzed by searching the information archived and coded by The First Affiliated Hospital of University of Science and Technology of China (Anhui Provincial Hospital) and Anhui Fuyang Second People’s Hospital, including gender, age, coexisting diseases, epidemiology, clinical symptoms, and peripheral oxygen saturations. Not all patients received relevant laboratory tests at a particular time, and we put emphasis on white blood cell count, lymphocytes percentage, CRP, and PCT. This study focused on the changes in body temperature, respiratory function, and CT findings before and after treatment with tocilizumab.

### Statistical Analysis.

All statistical data were analyzed by IBM SPSS software v.16.0 and are expressed as means ± SD. Paired *t* tests analyses have been used in Fig. 2 *B*–*D*. Data Sharing.

Requests for materials should be addressed to the corresponding authors.
